# The COVID-19 pandemic in Brazilian pregnant and postpartum women: results from the REBRACO prospective cohort study

**DOI:** 10.1038/s41598-022-15647-z

**Published:** 2022-07-11

**Authors:** Renato T. Souza, Jose G. Cecatti, Rodolfo C. Pacagnella, Carolina C. Ribeiro-Do-Valle, Adriana G. Luz, Giuliane J. Lajos, Guilherme M. Nobrega, Thayna B. Griggio, Charles M. Charles, Silvana F. Bento, Carla Silveira, Fernanda G. Surita, Maria J. Miele, Ricardo P. Tedesco, Karayna G. Fernandes, Sérgio H. A. Martins-Costa, Frederico J. A. Peret, Francisco E. Feitosa, Rosiane Mattar, Evelyn Traina, Edson V. Cunha Filho, Janete Vettorazzi, Samira M. Haddad, Carla B. Andreucci, José P. Guida, Mario D. Correa Junior, Marcos A. B. Dias, Leandro De Oliveira, Elias F. Melo Junior, Marília G. Q. Luz, Maria Laura Costa, REBRACO Study Group, Renato T. Souza, Maria Laura Costa, Jose G. Cecatti, Rodolfo C. Pacagnella, Carolina C. Ribeiro-do-Valle, Adriana G. Luz, Giuliane J. Lajos, Guilherme M. Nobrega, Thayna B. Griggrio, Charles M. Charles, Silvana F. Bento, Carla Silveira, Fernanda G. Surita, Maria J. Miele, Sherly Metelus, Lester Castro, Stephanie Pabon, Amanda D. Silva, Paulo S. R. Junior, Thais G. Sardinha, Rodolfo R. Japenga, Erica R. F. Urquiza, Maíra R. Machado, Marcela Maria Simões, Larissa M. Solda, Juliana Vasconcellos Freitas-Jesus, Rachel Esteves Soeiro, Ricardo P. Tedesco, Karayna G. Fernandes, Patrícia B. Peres, Cristiane L. Arbeli, Rafael M. Quevedo, Carolina F. Yamashita, Julia D. Corradin, Isabella Bergamini, Sérgio H. A. Martins-Costa, José Geraldo L. Ramos, Maria Lúcia R. Oppermann, Laisa S. Quadro, Lina Marins, Érika V. Paniz, Thaís Vicentini Xavier, Frederico J. A. Peret, Marina H. L. Almeida, Bruna F. V. Moura, Lidiane R. França, Hanna Vieira, Rafael B. Aquino, Aline C. Costa, Francisco E. Feitosa, Daisy Pinheiro, Denise Cordeiro, Priscila L. Miná, Carol Dornellas, Rosiane Mattar, Evelyn Traina, Sue Yazaki-Sun, Priscilla Mota, Arimaza C. Soares, Edson V Cunha Filho, Janete Vettorazzi, Ellen Machado, Anne Bergmann, Gustavo Raupp Santos, Samira M. Haddad, Aline Tosetto, Sabrina Savazoni, Carla B. Andreucci, Bruna E. Parreira, José P. Guida, Mario D. Correa Junior, Caio Leal, Rayra Amana, Marcos A. B. Dias, Marcos Nakamura-Pereira, Bruna O. Guerra, Gabriela Gorga, Leandro De Oliveira, Kevin F. A. Oliveira, Mariana Emi Varicoda Makyama, Elias F. Melo Junior, Débora F. Leite, Isabella Monteiro, Marília G. Q. Luz, Isabela R. Pereira, Clélia Andrade Salustrino, Valéria B. Pontes, Roberto Allen Silva Franco, João Paolo Bilibio, Gislânia P. F. Brito, Hana Paula C. Pinto, Danielle Leal Oliveira, Andrezza A. Guerra, Andrea O. Moura, Natasha Pantoja, Fernanda David, Alina Silva

**Affiliations:** 1 Department of Obstetrics and Gynecology, University of Campinas, 101 Alexander Fleming St, Campinas, SP Brazil; 2 Jundiaí School of Medicine—HU/FMJ, Jundiaí, SP Brazil; 3 Clinics Hospital of Porto Alegre, Porto Alegre, RS Brazil; 4 UNIMED Maternity—UNIMED/BH, Belo Horizonte, MG Brazil; 5 Federal University of Ceará—MEAC/UFC, Fortaleza, CE Brazil; 6 Federal University of São Paulo—UNIFESP/EPM, São Paulo, SP Brazil; 7 Moinhos de Vento Hospital—HMV, Porto Alegre, RS Brazil; 8 Jorge Rossmann Regional Hospital—Sócrates Guanaes Institute, Itanhaém, SP Brazil; 9 Federal University of São Carlos/UFSCAR, São Carlos, SP Brazil; 10 Sumaré State Hospital—HES, Sumaré, SP Brazil; 11 Federal University of Minas Gerais—HC/UFMG, Belo Horizonte, MG Brazil; 12 Fernandes Figueira Institute—IFF/Fiocruz, Rio de Janeiro, RJ Brazil; 13 São Paulo State University School of Medicine, Botucatu, SP Brazil; 14 Federal University of Pernambuco—HC/UFPE, Recife, PE Brazil; 15 Santa Casa de Misericórdia of Pará, Belém, PA Brazil

**Keywords:** Medical research, Epidemiology

## Abstract

Brazil presented a very high number of maternal deaths and evident delays in healthcare. We aimed at evaluating the characteristics of SARS-CoV-2 infection and associated outcomes in the obstetric population. We conducted a prospective cohort study in 15 Brazilian centers including symptomatic pregnant or postpartum women with suspected COVID-19 from Feb/2020 to Feb/2021. Women were followed from suspected infection until the end of pregnancy. We analyzed maternal characteristics and pregnancy outcomes associated with confirmed COVID-19 infection and SARS, determining unadjusted risk ratios. In total, 729 symptomatic women with suspected COVID-19 were initially included. Among those investigated for COVID-19, 51.3% (n = 289) were confirmed COVID-19 and 48% (n = 270) were negative. Initially (before May 15th), only 52.9% of the suspected cases were tested and it was the period with the highest proportion of ICU admission and maternal deaths. Non-white ethnicity (RR 1.78 [1.04–3.04]), primary schooling or less (RR 2.16 [1.21–3.87]), being overweight (RR 4.34 [1.04–19.01]) or obese (RR 6.55 [1.57–27.37]), having public prenatal care (RR 2.16 [1.01–4.68]), planned pregnancies (RR 2.09 [1.15–3.78]), onset of infection in postpartum period (RR 6.00 [1.37–26.26]), chronic hypertension (RR 2.15 [1.37–4.10]), pre-existing diabetes (RR 3.20 [1.37–7.46]), asthma (RR 2.22 [1.14–4.34]), and anaemia (RR 3.15 [1.14–8.71]) were associated with higher risk for SARS. The availability of tests and maternal outcomes varied throughout the pandemic period of the study; the beginning was the most challenging period, with worse outcomes. Socially vulnerable, postpartum and previously ill women were more likely to present SARS related to COVID-19.

## Introduction

The coronavirus disease 2019 (COVID-19) is an extremely transmissible and adaptative infection, and its spread was declared by WHO as pandemic in March 2020^1^. Awareness towards pregnant women and possible adverse outcomes were considered early on, due to previous experience with respiratory viruses^2,3^. Brazil, one of the countries most affected by the COVID-19 pandemic, presented a very high number of maternal deaths and evident delays in healthcare; reports from the national surveillance system on severe respiratory disease demonstrated a significant proportion of pregnant and postpartum women with confirmed infection, with no respiratory support or Intensive Care Unit (ICU) admission in the clinical management of COVID-19^4–6^. Delays in healthcare are clearly linked to adverse outcomes^7,8^, and settings that already face challenges in maternal and perinatal care are at most risk during a crisis. Especially this pandemic, which is no longer simply a sanitary crisis, but also economic, social and political has worsened the enormous disparities in Brazil^9–11^.

Lack of consistent information from low- and middle-income settings can be misleading and overlook the complete impact of the COVID-19 pandemic. The REBRACO cohort study aimed at evaluating the clinical features, severity and maternal and perinatal outcomes related to the COVID-19 infection in the Brazilian context. Also, we intended to investigate the characteristics of provision of care and conditions associated with poorer outcomes.

## Methods

The REBRACO initiative encompassed different methodological components (a cross-sectional study, a qualitative study, an ecological study, a cohort study and a crisis management committee in the COVID-19 Research Network) to broadly understand the impact of the COVID-19 pandemic on the obstetric population^12,13^. The current study represents the prospective multicenter cohort study conducted in 15 obstetric referral centers in four regions of Brazil. From 01 February 2020 to 28 February 2021, women with suspected COVID-19 infection who attended inpatient or outpatient health services at the participating centers were surveilled and invited to participate^13^. Eligibility criteria included pregnant or postpartum women who attended any obstetrical services of the participating centers presenting flu-like symptoms. The criteria for symptomatic COVID-19 infection were based on local protocols of infection surveillance in each center. The list of symptoms/signs is shown in the Table S1 (Supplementary Material). SARS-CoV-2 vaccination of pregnant and postpartum women began in May 2021 in Brazil—after the considered data collection period for the current study.

At enrolment, we collected information about sociodemographic, pregnancy, and medical history characteristics and on the initial clinical presentation of the supposed COVID-19. After the clinical presentation of a suspected case of COVID-19, the women were followed until pregnancy resolution if pregnant or until resolution of the COVID-19 suspected case if postpartum at admission. Data related to the suspicious symptomatic COVID-19 infection, characteristics of the management and resolution of the suspected infection, pregnancy and maternal and perinatal outcomes were retrieved for all women. Data were collected through review of medical records, telephone interviews with the women and/or in-person interviews. All personnel protection procedures were taken according to each hospital’s requirements for protection of both eligible women and research assistants. Study data were collected and managed using REDCap^®^^14^ electronic data capture tools hosted at CAISM/Unicamp server. The REDCap is a secure, web-based software platform designed to support data capture for research studies, providing (1) an intuitive interface for validated data capture; (2) audit trails for tracking data manipulation and export procedures; (3) automated export procedures for seamless data downloads to common statistical packages; and (4) procedures for data integration and interoperability with external sources. Research collaborators had hierarchical and clustered access to the system; data was properly anonymised and personal and contact information was kept confidential.

The COVID-19 diagnosis was confirmed based on the laboratory and/or radiologic pulmonary findings. Women were classified according to the status of COVID-19 infection: women with confirmed COVID-19 were defined as having any positive test for SARS-CoV-2 (either any rapid test or RT-qPCR) or a radiological finding of ground-glass opacities. Women who had a negative test (RT-PCR or rapid test) and did not have ground-glass opacities if submitted to radiological investigation were considered negative for COVID-19 infection; Women who were not tested and did not have ground-glass opacities if submitted to radiological investigation were considered not-tested. Figure S1 shows in detail cases considered confirmed for COVID-19 due to radiological criteria that were not previously confirmed by test results or not tested for SARS-CoV-2 (Supplementary Material).

For the evaluation of pregnancy outcomes, only women whose testing for COVID-19 was performed and follow-up was considered successful (childbirth information and COVID-19 status available) were included in the analysis. Women with late pregnancy outcome unavailable (unknown mode of delivery and gestational age at delivery) and postpartum women at enrolment were not considered.

### Statistical analysis and data management

We reported the number of women with suspected COVID-19 infection, the proportion of cases investigated (tests for COVID-19 performed) and cases confirmed for all participants in the whole period and further divided the considered time in three periods for the study: first period before May/15th/20, second period between May/15th/20 and Sep/01st/20 and third period after Sep/01st/20.

We compared sociodemographic, pregnancy and medical condition characteristics between women with confirmed and negative COVID-19, and those not-tested.

Then, we assessed the clinical features and severity of the flu-like disease, and estimated the risk for adverse pregnancy outcomes according to the COVID-19 status (confirmed vs negative COVID-19). Severity of COVID-19 infection included severe acute respiratory syndrome (SARS), admission to the intensive care unit (ICU), need for intubation and prone position, renal impairment, maternal death and any severe maternal outcomes, operationally defined as having any of the following: SARS, admission to NICU or maternal death. Pregnancy outcomes included preterm birth (any childbirth < 37 weeks), pre-eclampsia (new onset of hypertension, blood pressure higher or equal to 140/90 mmHg in two or more measures, after 20 weeks of gestation with proteinuria or other laboratorial or clinical signs of organ dysfunction), mode of delivery, adequacy of birth weight according to gestational age according to the GROW customised chart^15^, Apgar score below 7 at 5 min, neonatal respiratory distress, need for neonatal mechanical ventilation, admission to the neonatal intensive care unit (NICU), neonatal morbidity (any of the following: pneumonia, pulmonary dysplasia, intraventricular haemorrhage, convulsions, pulmonary haemorrhage, necrotizing enterocolitis, leukomalacia periventricular, retinopathy of prematurity and patent ductus arteriosus), congenital anomaly, neonatal death and any adverse perinatal outcomes according to the WHO Generic protocol (SARS-CoV-2 and pregnancy prospective cohort study), which included having any of the following: NICU admission, preterm birth, fetal death, neonatal death or miscarriage/abortion.

Also, we investigated risk factor for having SARS in women with confirmed COVID-19 using unadjusted relative ratios from a bivariate analysis. Finally, we estimated risk ratios for confirmed COVID-19 and for SARS in women with confirmed COVID-19 based on the symptoms presented at enrolment.

For comparisons using qualitative variables, Chi-squared or Fisher’s Exact tests were used when appropriate to asses statistical significance between groups. To determine the association of COVID-19 infection with pregnancy outcomes and risk factors for SARS, we estimated unadjusted relative ratios with 95% confidence intervals.

### Ethics statement

The study protocol followed the Declaration of Helsinki amended in Hong Kong in 1964 and it was approved by the Institutional Review Board (IRB) of the coordinating center (Letters of Approval numbers 4.047.168, 4.179.679, and 4.083.988). Also, the study was approved by the IRB of the School of Medical Sciences of the University of Campinas, Campinas/SP; IRB of the Jundiaí School of Medicine—HU/FMJ, Jundiaí/SP; IRB of the Clinics Hospital of Porto Alegre, Porto Alegre/RS; IRB of the UNIMED Maternity—UNIMED/BH, Belo Horizonte/MG; IRB of the Federal University of Ceará–MEAC/UFC, Fortaleza/CE; IRB of the Federal University of São Paulo—UNIFESP/EPM, São Paulo/SP; IRB of the Moinhos de Vento Hospital—HMV, Porto Alegre/RS; IRB of the Jorge Rossmann Regional Hospital—Sócrates Guanaes Institute, Itanhaém/SP; IRB of the Federal University of São Carlos/UFSCAR, São Carlos/SP; IRB of the Sumaré State Hospital—HES, Sumaré/SP; IRB of the Federal University of Minas Gerais–HC/UFMG, Belo Horizonte/MG; IRB of the Fernandes Figueira Institute—IFF/Fiocruz, Rio de Janeiro/RJ; IRB of the São Paulo State University School of Medicine, Botucatu/SP; IRB of the Federal University of Pernambuco—HC/UFPE, Recife/PE; IRB of the Santa Casa de Misericórdia of Pará, Belém/PA. This manuscript follows the STROBE Statement^16^. All women invited to participate received detail information about the study, the follow-up and the data and sample collections, when applicable. All included women provided an informed consent to their participation prior to be enrolled.

## Results

**Figure 1:**
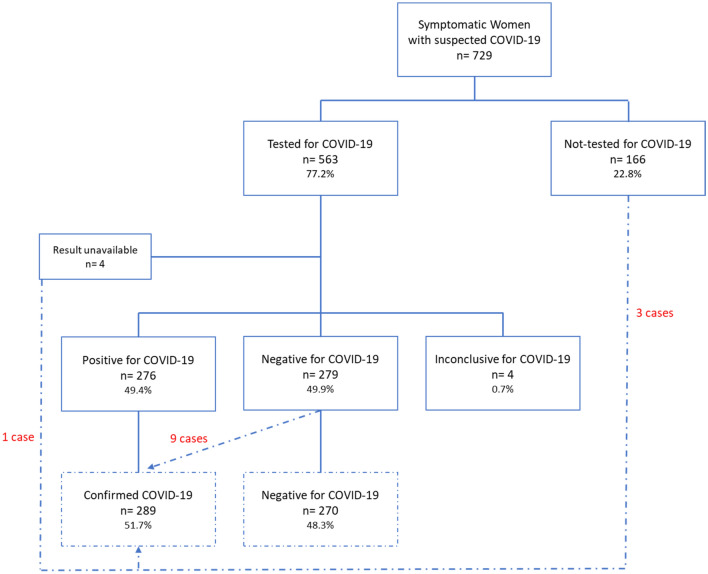
Flowchart of participants included in the REBRACO study according to test results and radiological criteria for confirmed and negative COVID-19 infection.

**Figure 2:**
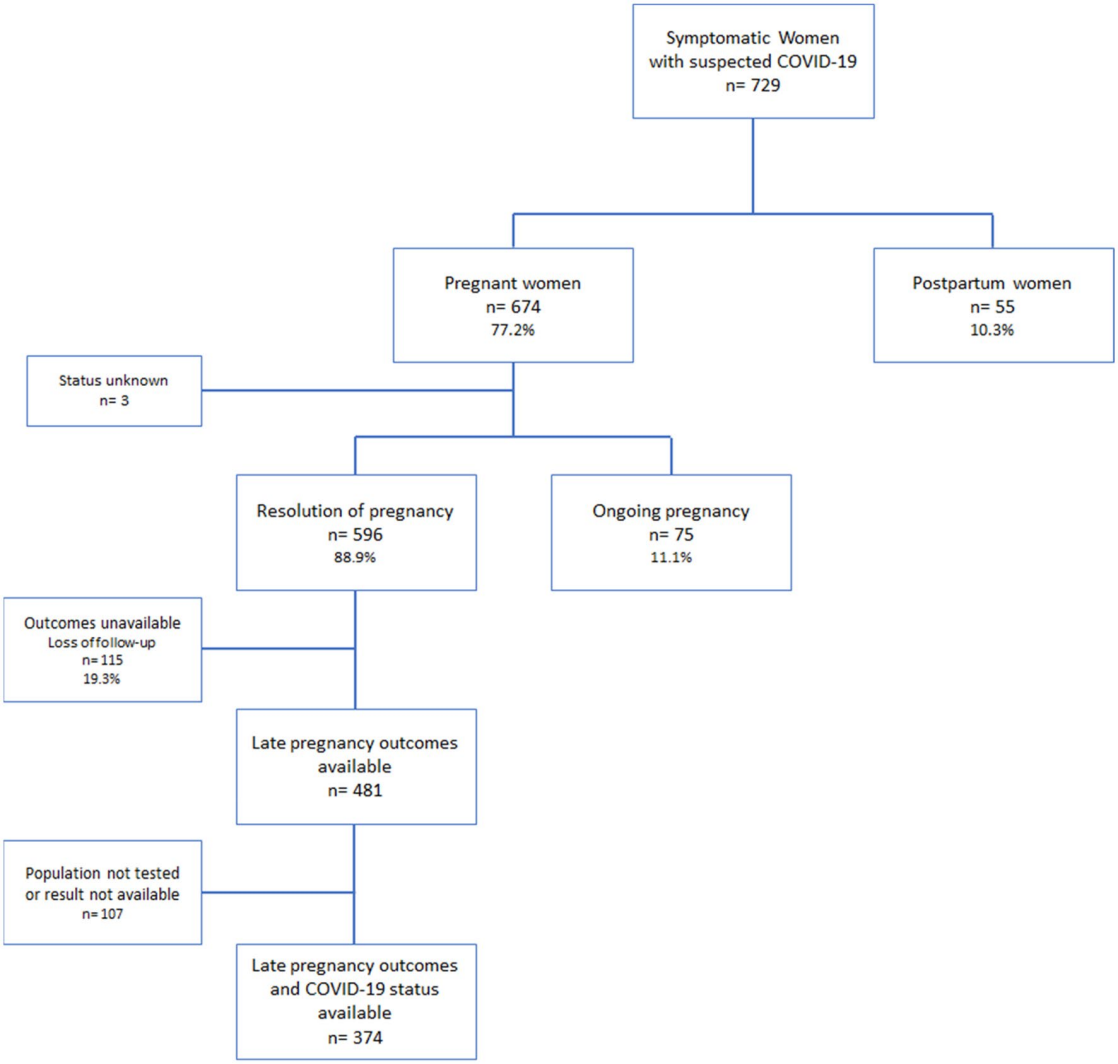
Flowchart of participants included in the REBRACO study according to the pregnancy status and outcomes availability.

**Figure 3:**
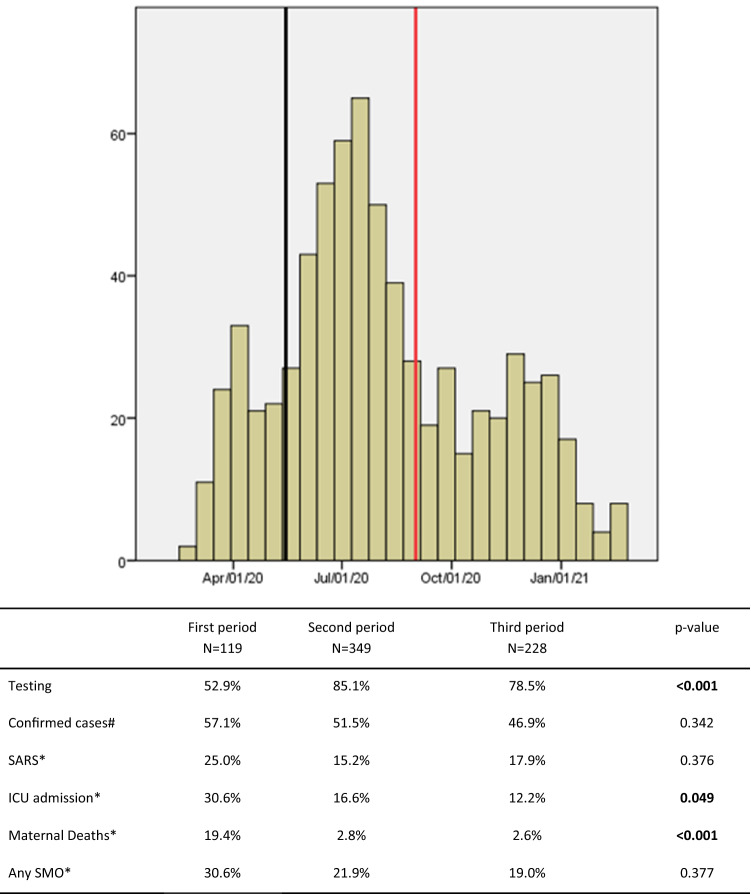
Case series of total included women divided by the three study periods.

The REBRACO study included 729 symptomatic women with suspected COVID-19 infection (flu-like syndrome) from Feb 2020 to Feb 2021, from which 77.2% (n = 563) were tested for SARS-CoV-2. After considering all investigations employed for COVID-19 (SARS-CoV-2 tests and/or COVID-19 radiological findings), 289 women (51.3%) were considered confirmed for COVID-19 and 270 were considered negative (48.3%) (Fig. 1). Figure 2 shows the enrolment and follow-up flowchart according to pregnancy outcomes. From the 729 women, 89.7% (n = 674) were pregnant and 10.3% (n = 55) were postpartum women at enrolment. Amongst all women who were supposed to have resolution of pregnancy until the end of the study period (n = 596), considering gestational age at enrolment, late pregnancy outcomes were available for 481 (80.7%) women and only 374 had also available data on COVID-19 infection status (Fig. 2). The total number of cases, proportion of the study population tested, the number of confirmed cases and related COVID-19 outcomes differed in the three study periods (Fig. 3). Initially (before May 15th), only 52.9% of the suspicious cases were tested and it was the period with the highest proportion of ICU admission and maternal deaths. The coverage of tests reached 85.1% and 78.5% of cases in the second (between May/15th/20 and Sep/01st/20 and third periods (after Sep/01st/20), respectively. Figure S1 shows that the peak of included cases was around July/2020, regardless of the status of COVID-19 infection (Supporting Information).

Table 1 shows the sociodemographic and obstetrical characteristics of women with suspected and confirmed SARS-CoV-2 infection. The women with 35 years or more, from the North/Northeast regions, with higher schooling, who had private and/or insurance coverage of health care, without chronic hypertension, and who used to smoke or drink alcohol were more likely to have confirmed COVID-19. The sociodemographic, pregnancy and maternal characteristics were also compared according to the status of testing (tested vs not tested for SARS-CoV-2) (Table S2; Supplementary Material). Women who were pregnant at the third trimester (54.3% vs 41.6%, p-value 0.006) and had chronic hypertension (10.7% vs 5.4%, p-value 0.043) were more likely to be tested for SARS-CoV-2. Table 2 shows clinical features and severity of infection according to the COVID-19 status. Women with confirmed COVID-19 were more likely to have more days of symptoms at enrolment, to have tachypnea and desaturation at admission, to be admitted to the ICU, to have SARS, to require intubation or prone position, to have renal impairment, death or any severe maternal outcomes; they were also less likely to have done multiple tests. Women who were not tested were less likely to have tachypnea or desaturation at admission, SARS, ICU admission or any severe maternal outcome (Table S2, S3; Supplementary Material).

**Table 1:** Sociodemographic and obstetrical characteristics of women with suspected and confirmed SARS-CoV-2 infection in the 15 REBRACO participating maternities.

Characteristics	Confirmed COVID-19 n = 289	Negative COVID-19 n = 270	Not-tested n = 163	p-value
**Age**				**0.001**
≤ 19	15 (5.2%)	35 (13.0%)	7 (4.3%)	
20–35	207 (71.6%)	194 (71.9%)	126 (77.3%)	
> 35	67 (23.2%)	41 (15.2%)	30 (18.4%)	
**Ethnicity**^**a**^				0.937
White	159 (55.8%)	149 (55.6%)	86 (57.3%)	
Non-White	126 (44.2%)	119 (44.4%)	64 (42.7%)	
**Region**				** < 0.001**
North/Northeast	45 (15.6%)	12 (4.4%)	11 (6.7%)	
Southeast	185 (64.0%)	230 (85.2%)	116 (71.2%)	
South	59 (20.4%)	28 (10.4%)	36 (22.1%)	
**Marital status**^**b**^				0.170
With partner	186 (65.7%)	150 (57.9%)	97 (61.0%)	
Without partner	97 (34.3%)	109 (42.1%)	62 (39.0%)	
**Schooling**^**c**^				** < 0.001**
None or Primary incomplete	20 (8.4%)	21 (9.3%)	9 (7.4%)	
Primary or Secondary	138 (57.7%)	171 (75.7%)	83 (68.0%)	
College or more	81 (33.9%)	34 (15.0%)	30 (24.6%)	
**Pre-pregnancy BMI**^**d**^				0.051
Underweight	2 (1.0%)	7 (4.4%)	0 (0.0%)	
Normal	60 (29.7%)	52 (32.9%)	40 (40.4%)	
Overweight	68 (33.7%)	41 (25.9%)	28 (28.3%)	
Obese	72 (35.6%)	58 (36.7%)	31 (31.3%)	
**Source of antenatal care**^**e**^				** < 0.001**
Public	186 (68.9%)	205 (83.7%)	108 (70.1%)	
Private/insurance/mixed	84 (31.1%)	40 (16.3%)	46 (29.9%)	
**Parity**^**f**^				0.143
0	102 (35.5%)	80 (30.0%)	66 (41.2%)	
1–2	128 (44.6%)	136 (50.9%)	63 (39.4%)	
≥ 3	57 (19.9%)	51 (19.1%)	31 (19.4%)	
Planned pregnancy^g^	141 (56.4%)	78 (45.3%)	59 (56.7%)	0.056
Multiple pregnancy^h^	10 (3.5%)	5 (1.9%)	6 (3.7%)	0.428
**Pregnancy status at enrolment**^**i**^				**0.027**
1st Trimester	36 (12.5%)	34 (12.6%)	31 (19.6%)	
2nd Trimester	73 (25.3%)	63 (23.5%)	56 (33.5%)	
3rd Trimester	158 (54.7%)	148 (55.0%)	65 (41.1%)	
Postpartum	22 (7.5%)	24 (8.9%)	9 (5.7%)	
Chronic hypertension	14 (4.8%)	29 (10.7%)	8 (4.9%)	**0.012**
Pre-existing diabetes	6 (2.1%)	8 (3.0%)	2 (1.2%)	0.483
Asthma	21 (7.3%)	23 (8.5%)	14 (8.6%)	0.825
Anemia	4 (1.4%)	2 (0.7%)	0 (0.0%)	0.292
HIV	3 (1.0%)	2 (0.7%)	2 (1.2%)	0.872
Chronic kidney disease	1 (0.3%)	1 (0.4%)	0 (0.0%)	0.745
Smoking	2 (0.7%)	22 (8.1%)	6 (3.7%)	** < 0.001**
Alcohol drinking	0 (0.0%)	7 (2.6%)	2 (1.2%)	**0.022**

Missing information for ^a^19, ^b^21, ^c^135, ^d^263, ^e^53, ^f^8, ^g^196, ^h^3, ^i^6.

Significant values are in bold.

**Table 2:** Clinical features and severity of SARS-CoV-2 infection of women according to the COVID-19 status during pregnancy or postpartum.

Clinical features and severity	Confirmed COVID-19 n = 289	Negative COVID-19 n = 270	p-value
**Number of days with symptoms before enrolment**^**a**^			**0.008**
1–3	126 (46.3%)	159 (59.6%)	
4–10	110 (40.4%)	88 (33.0%)	
> 10	36 (13.2%)	20 (7.5%)	
Multiple tests^b^	56 (19.6%)	98 (36.3%)	** < 0.001**
Tachypnea at admission (> 24 bpm)^c^	71 (27.3%)	43 (18%)	**0.013**
Desaturation at admission (< 95%)^d^	22 (8.2%)	9 (3.6%)	**0.027**
**Initial management**^**e**^			**0.005**
Discharge from ER	131 (45.5%)	116 (43.0%)	
Ward admission	99 (34.5%)	108 (40.0%)	
Labor ward	30 (10.0%)	38 (14.0%)	
ICU admission	28 (9.7%)	8 (3.0%)	
SARS^f^	47 (16.3%)	17 (6.3%)	** < 0.001**
ICU admission at any time^f^	48 (16.7%)	17 (6.3%)	** < 0.001**
Intubation^g^	18 (7.2%)	3 (1.2%)	**0.001**^**#**^
Prone position^h^	14 (5.6%)	0 (0%)	** < 0.001**^**#**^
Renal impairment (Cr > 1.1)^i^	17 (13.2%)	10 (9.8%)	0.428
Maternal death^j^	13 (4.7%)	0 (0%)	**0.001**^**#**^
Any severe maternal outcome	62 (21.5%)	23 (8.5%)	** < 0.001**

Chi-squared tests were applied for all comparisons, except those indicated with # (Fisher’s Exact test).

Missing information for ^a^ 20, ^b^3, ^c^60, ^d^ 39, ^e^1, ^f^2, ^g^57, ^h^59, ^i^328, ^j^48.

Significant values are in bold.

We estimated the relative ratios for pregnancy and maternal outcomes in women with confirmed COVID-19 compared to women with flu-like syndrome but negative investigation for COVID-19 (Table 3). Neonates born from pregnant women who had confirmed COVID-19 were more likely to be submitted to neonatal mechanical ventilation (RR 1.78 [1.50–2.11]), to have neonatal morbidity (RR 1.43 [1.11–2.11]), congenital anomaly (RR 1.36 [1.02–1.81]) and neonatal death (RR 1.68 [1.27–2.22]).

**Table 3:** Risk estimates for adverse pregnancy outcomes according to COVID-19 infection status in pregnant women (n = 374).

Pregnancy outcomes	Confirmed COVID-19 n = 198	Negative COVID-19 n = 176	RR [95%CI]
**Pregnancy outcome**^a^			
Miscarriage/Abortion/ectopic	0 (0%)	2 (1.1%)	–
Fetal Death	4 (2.0%)	2 (1.1%)	1.25 [0.70–2.23]
Live birth	193 (98.0%)	171 (97.8%)	Ref
Preterm birth^b^	59 (30.3%)	40 (23.7%)	1.16 [0.95–1.42]
Pre-eclampsia^c^	21 (10.8%)	23 (13.3%)	0.89 [0.64–1.23]
**Mode of delivery**			
Vaginal birth	70 (35.3%)	71 (40.3%)	Ref.
Elective C-section	100 (50.6%)	72 (40.9%)	1.17 [0.95–1.44]
Intrapartum C-section	28 (14.1%)	33 (18.8%)	0.92 [0.67–1.27]
**Adequacy of birth weight**^**d**^			
SGA	42 (22.5%)	24 (14.8%)	1.21 [0.97–1.50]
AGA	126 (67.4%)	114 (70.4%)	Ref.
LGA	19 (10.1%)	24 (14.8%)	0.84 [0.58–1.20]
Apgar < 7 at 5th minute^e^	9 (4.7%)	5 (3.4%)	1.15 [0.77–1.72]
Neonatal respiratory distress^f^	43 (23.6%)	32 (20.8%)	1.07 [0.85–1.35]
Neonatal mechanical ventilation^g^	25 (13.6%)	3 (1.9%)	**1.78 [1.50–2.11]**
NICU admission^h^	51 (27.3%)	34 (20.7%)	1.17 [0.95–1.44]
Any neonatal morbidity^i^	22 (13.8%)	9 (6.0%)	**1.43 [1.11–1.85]**
Congenital anomaly^#j^	14 (7.6%)	5 (3.4%)	**1.36 [1.02–1.81]**
Neonatal death^k^	7 (3.8%)	1 (0.6%)	**1.68 [1.27–2.22]**
Any APO/WHO*^a^	73 (37.1%)	53 (30.3%)	1.14 [0.94–1.39]

Missing information for ^a^2, ^b^10, ^c^7, ^d^25, ^e^35, ^f^38, ^g^28, ^h^23, ^i^66, ^j^41, ^k^24.

*APO: NICU admission, preterm birth, fetal death, neonatal death, miscarriage/abortion.

^#^From the 19 cases of congenital anomaly. 6 were tested for SARS-CoV-2 neonatal infection; all were negative.

Significant values are in bold.

Table 4 shows the relative ratios for SARS among women with confirmed COVID-19 according to maternal and pregnancy characteristics. The conditions associated with higher risk for SARS were non-white ethnicity (RR 1.78 [1.04–3.04]), primary schooling or less (RR 2.16 [1.21–3.87]), being overweight (RR 4.34 [1.04–19.01]) or obese (RR 6.55 [1.57–27.37]), having prenatal care at public system (RR 2.16 [1.01–4.68]), planned pregnancies (RR 2.09 [1.15–3.78]), onset of infection at postpartum period (RR 6.00 [1.37–26.26]), chronic hypertension (RR 2.15 [1.37–4.10]), pre-existing diabetes (RR 3.20 [1.37–7.46]), asthma (RR 2.22 [1.14–4.34]), and anemia (RR 3.15 [1.14–8.71]).

**Table 4:** Bivariate analysis for risk factors associated to severe acute respiratory syndrome (SARS) in women with confirmed COVID-19.

Characteristics	SARS n = 47	Not SARS n = 241	RR [95% CI]
**Number of days with symptoms at enrolment**^**a**^			
< 7	34 (72.3%)	162 (72.0%)	Ref.
≥ 7	13 (27.7%)	63 (28.0%)	0.95 [0.55–1.76]
**Age**			
≤ 19	4 (8.5%)	10 (4.1%)	2.04 [0.83–4.99]
19–35	29 (61.7%)	178 (73.9%)	Ref.
> 35	14 (29.8%)	53 (22.0%)	1.49 [0.84–2.65]
**Ethnicity**^**b**^			
White	19 (41.3%)	139 (58.4%)	Ref.
Non-White	27 (58.7%)	99 (41.6%)	**1.78 [1.04–3.04]**
**Regions**			
North/Northeast	11 (23.4%)	34 (14.1%)	1.65 [0.91–2.99]
Southeast/South	36 (76.6%)	207 (85.9%)	Ref.
**Marital status**^**c**^			
With partner	30 (65.2%)	156 (66.1%)	Ref.
Without partner	16 (34.8%)	80 (33.9%)	0.96 [0.55–1.68]
**Schooling**^**d**^			
Primary or less	16 (43.2%)	46 (22.9%)	**2.16 [1.21–3.87]**
Secondary or more	21 (56.8%)	155 (77.1%)	Ref
**Pre-pregnancy BMI**^**e**^			
Underweight	0 (0.0%)	2 (1.2%)	–
Normal	2 (7.1%)	57 (32.9%)	Ref.
Overweight	10 (35.7%)	58 (33.5%)	**4.34 [1.00–19.01]**
Obese	16 (57.2%)	56 (32.4%)	**6.55 [1.57–27.37]**
**Source of antenatal care**^**f**^			
Public	34 (82.9%)	152 (66.7%)	**2.16 [1.01–4.68]**
Private/Insurance/Mixed	7 (17.1%)	76 (33.3%)	Ref.
**Parity**^**g**^			
Primigravida	13 (28.3%)	88 (36.7%)	0.72 [0.39–1.30]
Multipara	33 (71.7%)	152 (63.3%)	Ref
Planned pregnancy^h^	24 (61.5%)	84 (40.0%)	**2.09 [1.15–3.78]**
Multiple pregnancy	0 (0%)	10 (4.1%)	–
**Pregnancy status at enrolment**			
1st Trimester	2 (4.2%)	34 (14.1%)	Ref.
2nd Trimester	12 (25.6%)	61 (25.3%)	2.95 [0.69–12.52]
3rd Trimester	26 (55.3%)	132 (54.8%)	2.96 [0.73–11.91]
Postpartum	7 (14.9%)	14 (5.8%)	**6.00 [1.37–26.26]**
Chronic hypertension	8 (17.0%)	17 (7.1%)	**2.15 [1.37–4.10]**
Pre-existing diabetes	3 (6.4%)	3 (1.2%)	**3.20 [1.37–7.46]**
Asthma	7 (14.9%)	14 (5.8%)	**2.22 [1.14–4.34]**
Anemia	2 (4.3%)	2 (0.8%)	**3.15 [1.14–8.71]**
HIV	0 (0%)	3 (1.2%)	–
Chronic kidney disease	0 (0%)	1 (0.4%)	–
Smoking	1 (2.1%)	1 (0.4%)	3.11 [0.75–12.74]

Missing information for ^a^16, ^b^4, ^c^6, ^d^50, ^e^87, ^f^19, ^g^2, ^h^39.

Significant values are in bold.

**Figure 4:**
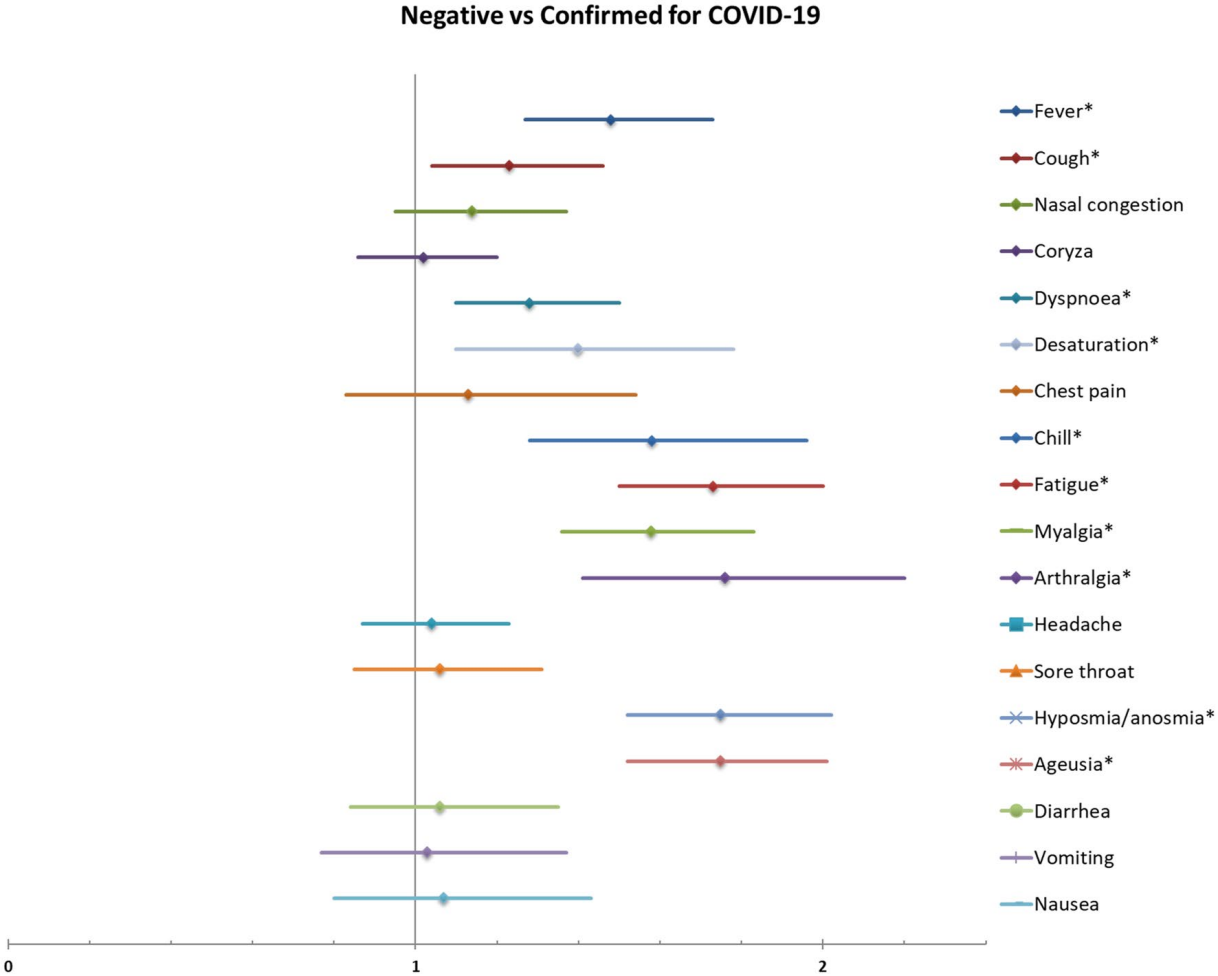
Risk ratios for confirmed COVID-19 in symptomatic women according to symptoms at enrolment.

**Figure 5:**
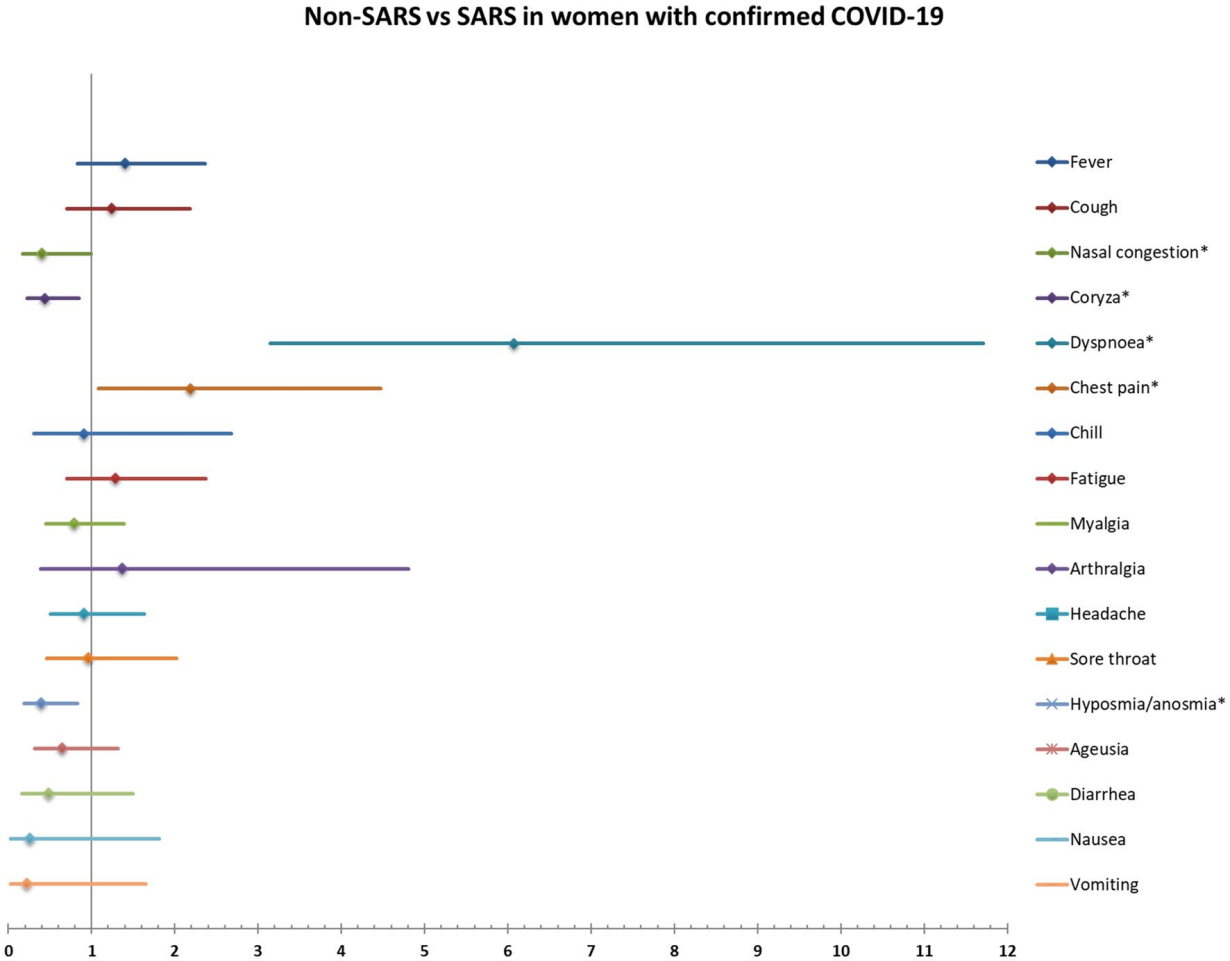
Risk ratios for SARS in women with confirmed COVID-19 according to symptoms at enrolment.

Figures 4 and 5 show the relative ratios for confirmed COVID-19 and for severe acute respiratory syndrome (SARS), respectively, in women with confirmed COVID-19 based on signs and symptoms at admission. Presenting fever, cough, dyspnea, desaturation, chills, fatigue, myalgia, arthralgia, hyposmia/anosmia and ageusia were significantly associated with confirmed COVID-19. The signs/symptoms significantly associated with higher risk for SARS in women with confirmed COVID-19 were dyspnea and chest pain. Nasal congestion, coryza, hyposmia/anosmia were associated with lower risk for SARS. Table S4 and S5 show the full list of relative ratios and respective 95% confidence intervals for each sign/symptom.

## Discussion

The REBRACO study was a comprehensive prospective epidemiological approach studying COVID-19 in pregnancy in Brazil. The initiative has established a multicentre network that performed evaluation and monitoring of maternal conditions related to COVID-19 in symptomatic pregnant and postpartum women, and also collected relevant information on healthcare to better plan actions related to confronting the pandemic in the participating centers.

Maternal and pregnancy outcomes from women who had COVID-19 seem to vary according to the context; women from low-income settings are more vulnerable to adverse outcomes due to COVID-19^17^ and underlying conditions such as asthma, non-white ethnicity, older age (> 34 years) and having over 35 weeks of gestation were factors independently associated to severe COVID-19^18^. According to our study, approximately one in six women with confirmed COVID-19 infection had SARS (16.3%) and required admission to the intensive care unit (16.7%). The lethality rate of COVID-19 was 4.7% in the obstetric population. Also, around a fifth of women had any severe maternal outcome which included SARS, admission to ICU or maternal death.

A secondary analysis of a multicentre international study involving 73 centers in 22 different countries showed that the incidence of composite adverse fetal outcome (abortion, stillbirth, neonatal death and perinatal death) was significantly higher when the infection occurred in the first trimester^19^. In our study, we found that postpartum women had six-fold increased risk for SARS compared to first trimester pregnant woman. Also, trimester of infection was not identified as a significant risk factor. However, we acknowledge that this is still a relevant subject of investigation, considering the possible impact on maternal–fetal interface and long-term consequences of the disease. A single center prospective cohort study conducted in Turkey including more than 1400 pregnant women showed that the infection’s course and obstetric consequences may change between pregnant trimesters. Deterioration or need for advanced support can be observed even in pregnant women with no other health issues^20^.

Furtheremore, special attention should be given to postpartum women, once they might be at risk for the first and second types of delays. The need for taking care of the baby, stress, new onset or exacerbation of mental health disorders and constraints of physiological needs may result in fatigue and sleep disruption may play a role on postponing their own care^21^. The INTERCOVID Multinational Cohort Study comprised of 43 centers in 18 countries showed that COVID-19 was associated with higher risk for preterm birth (1.59-fold), especially provider-initiated PTB (1.97-fold), low birth weight (1.5-fold) and severe neonatal morbidity (2.6-fold)^22^. Also, there are systematic reviews on the topic showing that severe outcomes are associated with the moment of pregnancy, presence of some coexisting morbidities and availability of local resources to early identify signs of severity in order to provide health support^23^.

A systematic review published in April, 2021, evaluated the differences of clinical presentation, management and prognosis of laboratory-confirmed COVID-19 between around 29,000 pregnant women and 560,000 non-pregnant women^24^. The risk of ICU admission (RR 2.26 [1.68–3.05]) and need of invasive mechanical ventilation (RR 2.68 [2.07–3.47]) were significantly higher amongst pregnant women. Although the controls (non-pregnant women) differed in age, obesity and smoking status, and ethnicity characteristics, the higher risk for adverse outcomes highlights the importance of adequate surveillance of cases involving pregnant women^24^. During pregnancy there are physiological changes involving the immunological systems (altered cell-and-antibody-mediated immune response), cardiovascular system (increase of maternal blood volume, heart rate, cardiac output by 30–50%, and vascular resistance decreases) and respiratory system (decrease in functional residual capacity, end-expiratory volumes, and residual volumes)^25^. These changes may explain why the risk of severe COVID-19 may be higher during pregnancy than in the general population.

In Brazil, data on maternal outcomes related to the COVID-19 pandemic suggest that the access and quality of health care for pregnant and postpartum women may have been neglected^5^. Our data has shown that vulnerable women (non-white, low schooling, attending ANC service only at public system) were more likely to present SARS. In another Brazilian study including 669 maternal COVID-19 SARS cases with similar age and morbidity, black women (n = 134) were more likely to be admitted with poorer health condition (higher prevalence of dyspnea and low O_2_ saturation at admission) and to have ICU admission (27.6% vs 19.4%, p < 0.001), mechanical ventilation (14.9% vs 7.3%, p < 0.001), and death (17.0% vs 8.9%, p < 0.001) than white women^5^. The involved underlying factors might include gender inequalities, racial disparities and defective policies involving general education and reproductive health^5,26^. A Brazilian study addressing the Acute Respiratory Distress Syndrome Surveillance System for COVID-19 cases among pregnant or postpartum women in early 2020 showed that black women were more likely to present severe COVID-19 infection and to die when compared to white women^5^. In addition, ICU or respiratory supports were not available for approximately 27% and 14%, respectively, of the women who had died due COVID-19^6^.

A cross-section study conducted in Jordan held telephone interviews with 1300 participants (men and women) to address gender-based disparities during COVID-19 including health indices, mental well-being and economic burden^27^. The study showed that women in Jordan are experiencing worse outcomes in terms of mental well-being and economic burden, which may widen the gender gap issue. Also, the access to antenatal care was available for only half of the Jordanian pregnant women interviewed. Not only the direct effect of the SARS-CoV-2 infection may be responsible for maternal and pregnancy outcomes, but the substantial effect of the pandemic on the health care services. A comprehensive systematic review assessed the impact of the COVID-19 pandemic on maternal and pregnancy outcomes. They included 40 studies from Jan, 2020 to Jan, 2021 and demonstrated that maternal and perinatal outcomes have worsened globally, especially in low-resource settings, which reinforced the need for policies to strengthen health care systems^28^.

Testing capacity can be considered an indirect indicator of the local policies favouring COVID-19 spread control. A study conducted in four regions of Italy in the early outbreak of the pandemic (Feb–Mar 2020) assessed the association between testing policies and COVID-19 mortality^29^. The study showed that regions that applied a broader testing policy had significant less COVID-19 mortality. Ideally, tests should have been offered for all women. According to the guidelines of the Brazilian´s Ministry of Health, RT-qPCR for universal screening at delivery or for symptomatic women should be offered for all pregnant and postpartum women^30^. Although the guideline has followed international recommendation as those given by the WHO general´s director (saying: “test, test, test”)^31^, it has never been actually implemented by the government. The testing provision and it´s use for promoting individual and collective counselling have been very heterogeneous and scarce across the country^32^. Also, Brazil for a long time lacked solid programmes in favour of pandemic-containment strategies. The country, which has about 3,000,000 deliveries/ year, faced conflicting outrageous policies against vaccines, massive testing and use of personal protective equipment by politicians^33^. Recently, an ecological study assessing country-level determinants associated with severity of COVID-19 in 37 countries excluded Brazil from the analysis of testing capacity due to lack of representative and reliable data^34^. According to our data, some few maternal characteristics were associated with the higher provision of SARS-CoV-2 tests, including being at third trimester pregnant or postpartum periods and history of chronic hypertension. Also, women who were tested were more likely to have tachypnea or desaturation at admission, SARS, ICU admission or any severe maternal outcome. Although it suggests that, due lack of resources, women at higher risk were more likely to have access to tests, the efforts should be taken to promote universal testing coverage among pregnant and postpartum women, not only for preventing morbidity but to corroborate recommendation related to the combat of the spread of the virus and to better follow-up the women. Our high positivity among suspected cases suggest that testing was mostly available for more severe cases, notably, in some institutions where testing was only performed if there was the need for hospital admission.

There are some risk-stratification and prediction models developed for non-pregnant population^35–37^, but it may not be applicable for the obstetric population due to the pregnancy physiological modifications. Our findings may be useful to inform the development of risk stratification coupled with specific strategies for managing healthcare. The calculation of risk ratios for confirmed COVID-19 and for SARS related to COVID-19 may be useful for developing models containing these symptoms, which can help in the identification and management of cases of COVID-19 in pregnant women, especially in contexts with low availability of diagnostic tests or provision of limited resources such as ICU beds. In our study, symptomatic women who were admitted to the ICU were more likely to have chronic conditions such as asthma (16.2% vs 7.0%, p-value = 0.007; data not shown), overweight or obesity (85.8% vs 64.2%, p-value = 0.017; data not shown), chronic hypertension (16.2% vs 8.8%, p-value = 0.049; data not shown) and confirmed COVID-19 (73.8% vs 48.6%, p-value < 0.001; data not shown) when compared to women who were not admitted to ICU.

Our definition for confirmed COVID-19 cases did not include only positive RT-PCR tested cases; it included both laboratory specific tests (RT-PCR, serology or antigen tests) and/or radiological findings. The nasopharyngeal RT-PCR is considered the gold-standard test for confirming SARS-CoV-2 exposure. However, an alternative definition based on other findings may be considered, especially in low-resourced settings. Considering that general laboratory findings and clinical presentation (symptoms and signs) are very unspecific in the COVID-19 infection^24,38^, the use of radiological findings (usually ground-glass opacities) may be a reasonable alternative for managing and treating patients with COVID-19 cases^39,40^. Despite the difficult access to CT scans, its findings have high positive predictive value and can be used as an alternative method to confirm the diagnosis. The Centers for Disease Control and Prevention (CDC/USA) and the Brazilian Health Regulatory Agency (ANVISA) have recommended the use of suggestive radiological findings in the definition of confirmed COVID-19 cases^41,42^.

In late 2020, there was raised awareness towards the possibility of worse outcomes associated to new SARS-CoV-2 variants of concern (VOCs) with reported increased transmissibility, risk of hospitalization and virulence^20,43^. The dissemination of VOCs in Brazil was reported since December 2020, mostly the Gamma lineage (PANGO: P.1). The Alpha lineage of SARS-CoV-2 (PANGO: B.1.1.7) was also introduced in Brazil early during 2021^44^.

The higher frequency of congenital anomaly in confirmed COVID-19 pregnant women rises concern, however, this might reflect the enhanced surveillance employed to positive COVID-19 cases and not the virus itself. From the 19 cases of congenital anomaly, only 6 were tested for SARS-CoV-2 neonatal infection; all were negative The Brazilian Teratology Information Service has proposed some strategies to investigate, detect and prevent possible embryonic damaging effects of the new coronavirus, including multidisciplinary approach to report the events^43^. Nevertheless, data from this national system has not been published yet; multicenter well-designed studies are crucial for addressing this topic.

This was a multicentre prospective study comprising 15 maternities in four regions of Brazil, including maternities with public, private and mixed maternities with deliveries per year ranging from 2000 to 6000 in the period. The study had a significant loss of follow-up, especially for pregnancy outcomes (19.3%). The majority of the participating centers were local/regional referral units for COVID-19 cases, but in most cases, they were not able to closely follow the women who had mild-infection and who did not require hospitalization. This should be taken into account during the interpretation of our findings, as it may have overestimated the rate of poorer outcomes for those who were followed until the end of pregnancy.

Our results suggest structural problems of access and quality of health services. Although COVID-19 is present in all social contexts, the pandemic highlighted the social discrepancies that worsening results of the disease in Brazil. COVID-19 infection in pregnancy results in increased maternal morbidity and mortality and need for management resources such as admission to the ICU. Proper surveillance, testing and follow-up of suspected cases and an appropriate structuring of obstetric units widely implemented are crucial for fighting the pandemic and reducing the burden to maternal health. The findings from this study may help to promote awareness about the situation and to increment policies for decreasing disparities among vulnerable populations.

## Supplementary Information

Supplementary Information.

## Data Availability

Considering that the REBRACO study group is still conducting ancillary analyses addressing other topics related to this initiative and that the Ethical approval given for the study did not take into account the public availability of the information, the data will be only available upon request and under revision by the Ethical Review Board.
