# Supplementary material for: The COVID-19 pandemic in Brazilian pregnant and postpartum women: results from the REBRACO prospective cohort study
Source: Sci Rep. 2022 Jul 11;12:11758. doi: 10.1038/s41598-022-15647-z (PMC9272878; doi:10.1038/s41598-022-15647-z)
Supplement: Supplementary file 1 — Supplementary Information. [file 41598_2022_15647_MOESM1_ESM.docx]

**Table of content**

| **Content** | **Page** |
| --- | --- |
| Figure S1. SARS-CoV-19 suspected and confirmed infection cases in the REBRACO participating maternities throughout the pandemic period (Feb 2020 - Feb 2021) | 2 |
| Table S1. Signs and symptoms considered for suspicion of COVID-19 infection | 3 |
| Table S2. Sociodemographic and obstetrical characteristics of women according to status of testing for SARS-CoV-2 infection | 4 |
| Table S3. Clinical features and severity of SARS-CoV-2 infection of women according to the status of the tests for COVID-19 during pregnancy or postpartum | 6 |
| Table S4. Relative risks for confirmed COVID-19 in symptomatic women according to symptoms at enrolment | 7 |
| Table S5. Relative risks for SARS in women with confirmed COVID-19 according to symptoms at enrolment | 8 |

**
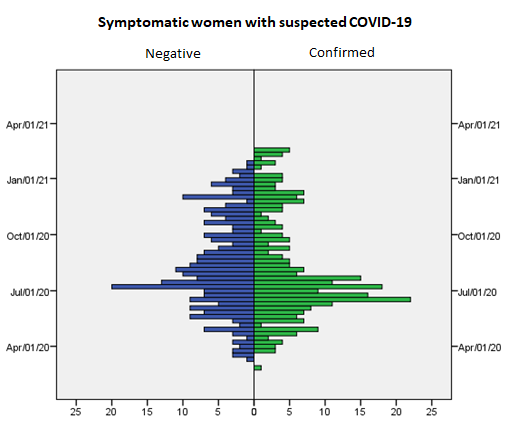

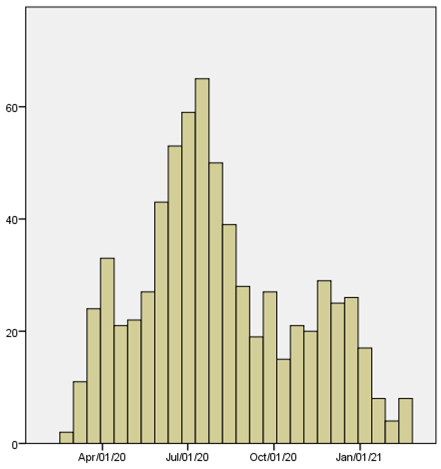
**

**Figure S1. SARS-CoV-19 suspected and confirmed infection cases in the REBRACO participating maternities throughout the pandemic period (Feb 2020 - Feb 2021)**

Legend: Histogram of the cases included in the REBRACO study from Feb/2020 to Feb/2021.

| **Table S1. Signs and symptoms considered for suspicion of COVID-19 infection** |
| --- |
| **Signs/Symptoms** |
| Fever |
| Cough |
| Nasal Congestion (Runny/stuffy nose) |
| Coryza |
| Dyspnea (Shortness of breath) |
| Chest pain |
| Chill |
| Diarrhea |
| Vomiting |
| Nauseas |
| Wheezing |
| Dizziness |
| Fatigue |
| Myalgia (Muscle pains) |
| Arthralgia |
| Headache |
| Sore throat |
| Hyposmia/Anosmia (Loss of smell) |
| Ageusia (Loss of taste) |
| Desaturation/Oxygen saturation <95% |
| Loss of conscious/confused |
| Convulsion |
| Cyanosis |
| Skin rash |
| Skin ulcer |
| Difficulty swallowing |
| Dehydration |
| Inappetence |
| Lower chest indrawing |
| Abdominal pain |
| Conjunctivitis |
| Lymphadenopathy |
| Contractions |
| Decreased fetal movements |
| Vaginal bleeding |
| Inability to walk |

| **Table S2. Sociodemographic and obstetrical characteristics of women according to status of testing for SARS-CoV-2 infection** | | | |
| --- | --- | --- | --- |
| **Characteristics** | **Tested**  **n=563** | **Not tested**  **n=163** | **p-value** |
| **Age** |  |  | 0.120 |
| ≤19 | 7 (4.2%) | 50 (8.9%) |  |
| 20-35 | 129 (77.7%) | 405 (71.9%) |  |
| >35 | 30 (18.1%) | 108 (19.2%) |  |
| **Ethnicity^a^** |  |  | 0.740 |
| White | 88 (57.5%) | 312 (56.0%) |  |
| Non-White | 65 (42.5%) | 245 (44.0%) |  |
| **Region** |  |  | 0.124 |
| North/Northeast | 11 (6.6%) | 57 (10.1%) |  |
| Southeast | 119 (71.7%) | 416 (73.9%) |  |
| South | 36 (21.7%) | 90 (16.0%) |  |
| **Marital Status^b^** |  |  | 0.999 |
| With partner | 100 (61.7%) | 337 (61.7%) |  |
| Without partner | 62 (38.3%) | 209 (38.3%) |  |
| **Schooling^c^** |  |  | 0.850 |
| None or Primary incomplete | 9 (7.2%) | 41 (8.8%) |  |
| Primary or Secondary | 84 (67.2%) | 310 (66.4%) |  |
| College or more | 32 (25.6%) | 116 (24.8%) |  |
| **Pre-pregnancy BMI^d^** |  |  | 0.097 |
| Underweight | 0 (0%) | 9 (2.5%) |  |
| Normal | 42 (41.6%) | 110 (30.6%) |  |
| Overweight | 28 (27.7%) | 109 (30.4%) |  |
| Obese | 31 (30.7%) | 131 (36.5%) |  |
| **Source of antenatal care^e^** |  |  | 0.190 |
| Public | 110 (70.5%) | 393 (75.7%) |  |
| Private/Insurance/Mixed | 46 (29.5%) | 126 (24.3%) |  |
| **Parity^f^** |  |  | 0.132 |
| 0 | 67 (41.1%) | 186 (33.3%) |  |
| 1-2 | 64 (39.3%) | 265 (47.5%) |  |
| ≥3 | 32 (19.6%) | 107 (19.2%) |  |
| **Planned pregnancy^g^** | 59 (55.1%) | 225 (52.9%) | 0.684 |
| **Multiple pregnancy^h^** | 6 (3.6%) | 16 (2.9%) | 0.605 |
| **Pregnancy status at enrolment^i^** |  |  | **0.006** |
| 1st Trimester | 31 (19.3%) | 71 (12.6%) |  |
| 2nd Trimester | 54 (33.5%) | 140 (24.9%) |  |
| 3rd Trimester | 67 (41.6%) | 305 (54.3%) |  |
| Postpartum | 9 (5.6%) | 46 (8.2%) |  |
| **Chronic hypertension** | 9 (5.4%) | 60 (10.7%) | **0.043** |
| **Pre-existing diabetes** | 2 (1.0%) | 14 (2.5%) | 0.322 |
| **Asthma** | 14 (8.4%) | 44 (7.8%) | 0.796 |
| **Anemia** | 0 (0%) | 7 (1.2%) | 0.149 |
| **HIV** | 2 (1.2%) | 5 (0.9%) | 0.713 |
| **Chronic kidney disease** | 0 (0%) | 2 (0.4%) | 0.442 |
| **Smoking** | 6 (3.6%) | 24 (4.3%) | 0.712 |
| **Alcohol drinking** | 2 (1.2%) | 7 (1.2%) | 0.968 |

Missing information for a) 1, b) 3, c) 11, d) 4, e) 8, f) 16, g) 14, h) 39, i) 13.

*APO: NICU admission, preterm birth, fetal death, neonatal death, miscarriage/abortion.

| **Table S3. Clinical features and severity of SARS-CoV-2 infection of women according to the status of the tests for COVID-19 during pregnancy or postpartum** | | | |
| --- | --- | --- | --- |
| **Clinical features and severity** | **Tested**  **n= 559** | **Not tested**  **n=163** | **p-value** |
| **Number of days with symptoms before enrolment^a^** |  |  | 0.121 |
| 1-3 | 285 (52.9%) | 97 (61.0%) |  |
| 4-10 | 198 (36.7%) | 52 (32.7%) |  |
| >10 | 56 (10.4%) | 10 (6.3%) |  |
| **Tachypnea at admission^b^** | 114 (22.8%) | 19 (12.8%) | **0.008** |
| **Desaturation at admission (oximetry <95%)^c^** | 31 (6.0%) | 2 (1.4%) | **0.022** |
| **Initial management^d^** |  |  | **<0.001** |
| Discharge from ER | 247 (44.3%) | 115 (71.4%) |  |
| Ward admission | 207 (37.1%) | 26 (16.1%) |  |
| Labor ward | 68 (12.2%) | 17 (10.6%) |  |
| ICU admission | 36 (6.5%) | 3 (1.9%) |  |
| **SARS^e^** | 64 (11.5%) | 6 (3.8%) | **0.004** |
| **ICU admission at any time^d^** | 65 (11.7%) | 3 (1.9%) | **<0.001** |
| **Intubation^f^** | 21 (4.2%) | 2 (1.6%) | 0.162 |
| **Prone position^g^** | 14 (2.8%) | 1 (0.8%) | 0.188 |
| **Renal impairment (Cr>1.1)^h^** | 27 (11.7%) | 1 (5.6%) | 0.428 |
| **Maternal death^i^** | 13 (2.5%) | 1 (0.8%) | 0.216 |
| **Any severe maternal outcome^j^** | 85 (15.2%) | 7 (4.3%) | **<0.001** |

Missing information for a) 25, b) 75, c) 54, d) 3, e)5, f) 93, g) 96, h) 473, i) 81, j) 2.

| **Table S4. Relative risks for confirmed COVID-19 in symptomatic women according to symptoms at enrolment** | |
| --- | --- |
| **Signs/Symptoms** | **Relative Risk (95% CI)** |
| Fever* | 1.48 (1.27-1.73) |
| Cough* | 1.23 (1.04-1.46) |
| Nasal congestion | 1.14 (0.95-1.37) |
| Coryza | 1.02 (0.86-1.20) |
| Dyspnoea* | 1.28 (1.10-1.50) |
| Desaturation* | 1.40 (1.10-1.78) |
| Chest pain | 1.13 (0.83-1.54) |
| Chill* | 1.58 (1.28-1.96) |
| Fatigue* | 1.73 (1.50-2.00) |
| Myalgia* | 1.58 (1.36-1.83) |
| Arthralgia* | 1.76 (1.41-2.20) |
| Headache | 1.04 (0.87-1.23) |
| Sore throat | 1.06 (0.85-1.31) |
| Hyposmia/anosmia* | 1.75 (1.52-2.02) |
| Ageusia* | 1.75 (1.52-2.01) |
| Diarrhea | 1.06 (0.84-1.35) |
| Vomiting | 1.03 (0.77-1.37) |
| Nausea | 1.07 (0.80-1.43) |

| **Table S5. Relative risks for SARS in women with confirmed COVID-19 according to symptoms at enrolment** | |
| --- | --- |
| **Signs/Symptoms** | **Relative Risk (95% CI)** |
| Fever | 1.40 (0.83-2.36) |
| Cough | 1.24 (0.70-2.18) |
| Nasal congestion* | 0.41 (0.17-0.99) |
| Coryza* | 0.44 (0.23-0.85) |
| Dyspnoea* | 6.07 (3.15-11.71) |
| Chest pain* | 2.19 (1.08-4.47) |
| Chill | 0.91 (0.31-2.68) |
| Fatigue | 1.29 (0.70-2.37) |
| Myalgia | 0.79 (0.45-1.39) |
| Arthralgia | 1.37 (0.39-4.81) |
| Headache | 0.91 (0.51-1.63) |
| Sore throat | 0.97 (0.46-2.02) |
| Hyposmia/anosmia* | 0.40 (0.19-0.83) |
| Ageusia | 0.65 (0.32-1.32) |
| Diarrhea | 0.49 (0.16-1.50) |
| Nausea | 0.26 (0.03-1.81) |
| Vomiting | 0.23 (0.03-1.65) |
